# Nutrition support for HIV-TB co-infected adults in Senegal, West Africa: A randomized pilot implementation study

**DOI:** 10.1371/journal.pone.0219118

**Published:** 2019-07-18

**Authors:** Noelle A. Benzekri, Jacques F. Sambou, Ibrahima Tito Tamba, Jean Philippe Diatta, Ibrahima Sall, Ousseynou Cisse, Makhtar Thiam, Gaetan Bassene, Ndeye Maguette Badji, Khadim Faye, Fatima Sall, Jean Jacques Malomar, Moussa Seydi, Geoffrey S. Gottlieb

**Affiliations:** 1 Department of Medicine, University of Washington, Seattle, WA, United States of America; 2 Centre de Santé de Ziguinchor, Ziguinchor, Senegal; 3 Centre de Santé de Bignona, Bignona, Senegal; 4 Services des Maladies Infectieuses et Tropicales, Centre Hospitalier National Universitaire de Fann, Dakar, Senegal; 5 Department of Global Health, University of Washington, Seattle, WA, United States of America; São Paulo Municipal Health Department, BRAZIL

## Abstract

**Background:**

Food insecurity can contribute to poor adherence to both tuberculosis treatment and HIV antiretroviral therapy (ART). Interventions that target food insecurity have the potential to increase treatment adherence, improve clinical outcomes, and decrease mortality. The goals of this study were to compare the feasibility, acceptability, and potential impact of implementing two different forms of nutrition support for HIV-TB co-infected adults in the Casamance region of Senegal.

**Methods:**

We conducted a randomized pilot implementation study among HIV-TB co-infected adults initiating treatment for TB (ClinicalTrials.gov Identifier: NCT03711721). Subjects received nutrition support in the form of a local food basket or Ready-to-Use Therapeutic Food (RUTF), distributed on a monthly basis for six months.

**Results:**

A total of 178 monthly study encounters were completed by 26 HIV-TB co-infected adults; 14 received food baskets and 12 received RUTF. For both the food basket and RUTF, 100% of subjects obtained the supplement at every study encounter, transferred the supplement from the clinic to their household, and consumed the supplement. The food basket had greater acceptability and was more likely to be shared with members of the household. Adherence to TB treatment and ART exceeded 95%, and all outcomes, including CD4 cell count, hemoglobin, nutritional status, and food security, improved over the study period. All subjects completed TB treatment and were smear negative at treatment completion. The total cost of the local food basket was approximately $0.68 per day versus $0.99 for the RUTF.

**Conclusion:**

The implementation of nutrition support for HIV-TB co-infected adults in Senegal is feasible and may provide an effective strategy to improve adherence, treatment completion, and clinical outcomes for less than 1 USD per day. Further studies to determine the impact of nutrition support among a larger population of HIV-TB co-infected individuals are indicated.

## Introduction

Tuberculosis (TB) remains a leading cause of death in Senegal, West Africa [1] and poor adherence to treatment is a barrier to effective TB control [2]. Treatment interruptions are common and cure rates remain low, especially among individuals co-infected with HIV [3–5]. Food insecurity has been identified as a critical barrier to adherence and retention in care for both HIV and tuberculosis, especially in sub-Saharan Africa (SSA) [6–10]. Interventions that target food insecurity have the potential to improve treatment adherence and thus decrease transmission and mortality [6–11].

Prior to integrating nutrition support interventions into national strategies, the feasibility and acceptability of implementing different forms of nutrition support require evaluation. This information is needed by programs in order to determine which interventions to implement in a given setting. The Casamance region of Senegal has the highest burden of food insecurity in the country [12], with a prevalence of severe food insecurity among people living with HIV reaching 75% [13]. The goals of this study were to compare the feasibility, acceptability, and potential impact of implementing two different forms of nutrition support for HIV-TB co-infected adults in the Casamance.

## Methods

Participant recruitment and follow-up were conducted from June 28, 2016 to August 11, 2017 at the Centre de Santé de Ziguinchor and the Centre de Santé de Bignona, located in the Casamance region of Senegal, West Africa. All HIV-infected individuals ≥18 years of age, initiating treatment for tuberculosis through the Programme National de Lutte contre la Tuberculose, were eligible for inclusion. Eligible individuals were identified by healthcare personnel and referred to the social worker for informed consent and enrollment. Informed consent and all study encounters were conducted in the participant’s preferred language, including French, Wolof, Diola, Mandinka, Peular, or Creole. Study procedures were approved by the University of Washington Institutional Review Board and the Senegal Comité National d'Ethique pour la Recherche en Santé. Study encounters took place each month and included an enrollment encounter, referred to as M0, followed by six follow-up encounters, for a total of seven encounters per subject. Reimbursement was provided for the cost of round-trip transportation to each study encounter based upon the standard cost of public transportation from the zone of residence.

Participants were randomized to receive nutrition support in the form of either a food basket or Ready-to-Use Therapeutic Food (RUTF), distributed on a monthly basis for six months. Randomization was conducted by the social worker using an opaque envelope containing an equal number of assignment cards for each of the two forms of nutrition support. Each subject blindly selected one card from the envelope to determine if they were randomized to the food basket group or the RUTF group. Cards were selected without replacement. The components of the food basket were chosen based upon interviews conducted with 95 community members to identify dietary practices and locally preferred foods [14]. The monthly food basket consisted of 5kg of cowpeas (*Vigna unguiculata*) and 3kg of rice grown in Senegal, 1.5L of vegetable oil, and 1.6kg of powdered milk, which would provide an average of approximately 1200 kilocalories per day if consumed by one person during the course of one month. The RUTF (Plumpy’Nut produced by Nutriset, France) consisted of a micronutrient fortified peanut-based paste that could be consumed directly from individually wrapped sachets. The monthly RUTF ration consisted of 60 sachets, which would provide an average of 1000 kilocalories per day if consumed by one person during the course of one month. To decrease the possibility of stigmatization, both forms of nutrition support were provided in opaque containers to conceal the contents, and were distributed from a neighboring building separate from clinical care and other patients.

WHO staging was performed at enrollment [15]. Medical records and pharmacy records were reviewed to determine clinical history, HIV type, history of antiretroviral therapy (ART), ART regimen, use of co-trimoxazole, date of TB diagnosis, treatment regimen, and dates of treatment initiation and completion. Medication adherence was assessed monthly. Measures of adherence included patient-reported number of missed doses in the past 7 days, patient reported number of missed doses in the past 4 weeks, and medication possession (number of days of medication dispensed at last refill / number of days between date of last refill and date of study encounter). For each subject, overall adherence was determined by calculating the mean monthly adherence over the 6 month study period.

Participant height and weight were measured at each encounter. Malnourished was defined as a Body Mass Index (BMI) <18.5. History of weight loss was assessed using medical records and patient report. Per WHO guidelines, unexplained weight loss >10% of presumed or measured body weight was classified as a WHO stage 3 condition [15]. The Household Food Insecurity Access Scale (HFIAS) was used to determine household food insecurity status at enrollment, month 3 and month 6 [16]. The HFIAS is a 9-item questionnaire which provides a household food insecurity score on a scale of 1–4, with 1 being not food insecure, 2 being mildly food insecure, 3 being moderately food insecure, and 4 being severely food insecure. A HFIAS score of 2–4 was considered food insecure and a HFIAS of 4 was considered severely food insecure.

CD4 cell count, hemoglobin levels were measured at enrollment and month 6. CD4 cell counts were measured in Ziguinchor using the FACSCount analyzer (Becton Dickinson). Sputum smear microscopy for the detection of acid-fast bacilli (AFB) using the Ziehl-Neelsen technique and the Xpert MTB/RIF test (Cepheid) on sputum was performed at initiation and completion of tuberculosis treatment according to the protocol of the Programme National de Lutte contre la Tuberculose. AFB sputum culture was not available. Results were obtained from review of the medical record.

Subjects completed interviewer-administered questionnaires performed by the social worker to capture sociodemographic characteristics and assess uptake, acceptability, practices, and perceptions of the intervention. The questionnaires includes both closed and open ended questions. Responses to questions were translated into French and recorded on paper study forms by the social worker during the interview.

The costs of the food basket and local transportation were calculated in West African francs (XOF) and converted to U.S. dollars (USD) using an exchange rate of 602.5 XOF to 1 USD. The costs of the RUTF and shipping from France to Senegal were calculated in Euros (EUR) using an exchange rate of 0.884 Euros to 1 USD. Values were rounded to the nearest hundredth of a dollar. Chi-square and Fisher’s Exact tests were used to identify differences in baseline characteristics and outcomes among individuals randomized to receive RUTF versus those in the food basket group. The t-test was used to identify differences in means between groups and the Mann–Whitney U test was used to identify differences in medians between groups. Descriptive analysis was performed for all variables. The interquartile range was determined for continuous variables. Missing data were excluded from analysis. P-values <0.05 were considered significant. Data were analyzed using SPSS Statistics 23 (IBM, Armonk, N.Y.).

This study does not meet criteria for an Applicable Clinical Trial under 42 CFR 11.22(b) for Clinical Trials thus registration was not required prior to enrolment. This study was voluntarily registered retrospectively (ClinicalTrials.gov Identifier: NCT03711721). The authors confirm that all ongoing and related trials for this intervention have been voluntarily registered.

## Results

A total of 178 monthly study encounters were completed by 26 HIV-TB co-infected adults; 14 received food baskets and 12 received RUTF **(Table 1)**. Twenty-four participants attended all scheduled monthly follow-up visits and 2 participants missed scheduled follow-up visits due to travel outside the region. The mean number of days between study visits was 34.3. There was no loss to follow-up. All 26 participants completed TB treatment.

**Table 1:** Baseline characteristics of study participants in Ziguinchor and Bignona, Senegal, 2016–2017.

	All subjects n (%)	RUTF n (%)	Food basket n (%)	p-value
**Number of participants, N**	26	12	14	
**Site**				1.00
Ziguinchor	13 (50.0)	6 (50.0)	7 (50.0)	
Bignona	13 (50.0)	6 (50.0)	7 (50.0)	
**Age, median years (IQR)**	46 (29–55)	49 (34–55)	40 (26–53)	0.50
**Female**	13 (50.0)	6 (50.0)	7 (50.0)	1.00
**Education**				0.96
None	8 (33.3)	3 (30.0)	5 (35.7)	
Primary school	9 (37.5)	4 (40.0)	5 (35.7)	
Secondary school	7 (29.2)	3 (30.0)	4 (28.6)	
**Unemployed**	22 (84.6)	10 (83.3)	12 (85.7)	1.00
**Household (HH) size, median (IQR)**	11 (7–16)	10 (7–17)	13 (7–16)	0.57
HH members age <5, median (IQR)	2 (0–3)	2 (1–2)	2 (0–3)	0.89
HH members age ≥18, median (IQR)	8 (5–11)	7 (5–10)	8 (5–12)	0.55
**All household members unemployed**	15 (57.7)	8 (66.7)	7 (50.0)	0.39
**Clinic transportation time, round trip (minutes), median (IQR)**	60 (30–120)	75 (30–120)	60 (28–120)	0.46
**Clinic transportation expenditure, round trip median (IQR)**	$1.66 ($0.66 -$1.99)	$1.66 ($0.33-$1.99)	$1.66 ($0.66-$2.24)	0.77
**Daily household food expenditure, median (IQR)**	$1.66 ($0.83 -$4.15)	$1.66 ($0.83 -$4.15)	$2.49 ($0.83 -$4.36)	0.74
**HIV type**				0.67
HIV-1	19 (73.1)	8 (66.7)	11 (78.6)	
HIV-2	7 (26.9)	4 (33.3)	3 (21.4)	
**History of WHO stage 3 or 4 conditions**				
Fever >1m	9 (34.6)	4 (33.3)	5 (35.7)	1.00
Weight loss >10%	26 (100)	12 (100)	14 (100)	-
Diarrhea >1m	7 (26.9)	1 (8.3)	6 (42.9)	0.08
Pulmonary TB	26 (100)	12 (100)	14 (100)	-
**Co-trimoxazole**	17 (89.5)	9 (90.0)	8 (88.9)	1.00
**On ART before enrollment**	14 (56.0)	5 (45.5)	9 (64.3)	0.44
**ART regimen**				0.59
TDF + 3TC + EFV	17 (65.4)	7 (58.3)	10 (71.4)	
Triple NRTI	5 (19.2)	3 (25.0)	2 (14.3)	
LPV/r-based regimen*	3 (11.5)	1 (8.3)	2 (14.3)	
TDF + 3TC + NVP*	1 (3.8)	1 (8.3)	0 (0)	
**TB diagnosis**				0.72
Both sputum smear and Xpert positive	7 (26.9)	4 (33.3)	3 (21.4)	
Positive sputum smear only	12 (46.2)	6 (50.0)	6 (42.9)	
Positive Xpert only	4 (15.4)	1 (8.3)	3 (21.4)	
Clinical	3 (11.5)	1 (8.3)	2 (14.3)	
**Directly Observed Therapy (DOT)**				0.14
No DOT	15 (57.7)	6 (66.7)	9 (64.3)	
DOT by health-care worker	2 (7.7)	0 (0)	2 (14.3)	
DOT by family member	6 (23.1)	3 (33.3)	3 (21.4)	
Not documented	3 (11.5)	3 (25.0)	0 (0)	
**TB treatment regimen: 2HRZE/4HR**	24 (92.3)	11 (91.7)	13 (92.9)	0.54
**Days between TB diagnosis and TB treatment initiation, median (IQR)**	0 (0–1.25)	0 (0–1.50)	0 (0–1.50)	0.95

*The co-administration of rifampin with nevirapine or protease-inhibitors is not recommended.

Half of the subjects were enrolled at the Ziguinchor site and half were enrolled at the Bignona site. The median age of participants was 46 years (IQR 29–55) and half were female. One third had no formal education. The median household size was 11 (IQR 7–16). The majority of subjects (85%) were unemployed and more than half (58%) lived in households where all household members were unemployed.

The median round trip travel time to clinic was 60 minutes (IQR 30–120). The median round trip transportation expenditure was $1.66 (IQR $0.66-$1.99). The median expenditure for clinic transportation was equal to the median reported expenditure on daily food for the household.

The majority of subjects were infected with HIV-1 and all had WHO stage 3 or 4 disease. All subjects had pulmonary TB. Nearly 90% of subjects were receiving co-trimoxazole, none were receiving isoniazid preventive therapy (IPT), and 56% were receiving ART prior to enrollment. Among those receiving ART prior to enrollment, ART duration was available for 10 subjects. The median time on ART was 11 days (IQR 6–1371). The most common ART regimen was tenofovir/lamivudine/efavirenz (TDF/3TC/EFV). Four subjects were receiving ART regimens that are not recommended during treatment with rifampicin.

Seven subjects were diagnosed by both positive sputum smear and positive Xpert, 12 were diagnosed by positive sputum smear only, 4 were diagnosed by positive Xpert only, and 3 were diagnosed clinically. Of the 26 subjects, 23 had evaluation of sputum smears performed at M0, of which 19 (82.6%) were positive for AFB. Among the 19 subjects with sputum smear positive for AFB, 7 had positive Xpert results, 1 had negative Xpert results, and for 11 Xpert was not conducted. Fourteen subjects had Xpert performed at M0, of which 11 (78.6%) were positive. Among the 11 subjects with positive Xpert, 7 had positive sputum smear results, 1 had negative smear results, and for 3 sputum smear was not conducted. Rifampicin resistance was not found in any subjects. Among the 3 subjects who were diagnosed clinically, all had negative sputum smear results and 2 had negative Xpert results.

Fifteen subjects (58%) had no form of DOT; 2 subjects (8%) had DOT administered by a healthcare worker and 6 subjects (23%) had DOT administered at home by a family member. Subjects were treated according to the World Health Organization Guidelines for Treatment of Tuberculosis using isoniazid (H), rifampicin (R), pyrazinamide (Z), and ethambutol (E) [17]. The majority of subjects received TB treatment with 2HRZE/4HR. Two subjects were being retreated and received streptomycin (S) (2SHRZE/1HRZE/5HRE). The median time between TB diagnosis and treatment initiation was 0 days (IQR 0–1.25; range 0–3).

### Costs

The total cost of the RUTF was $0.99 per day, including shipping **(Table 2)**. The cost of the RUTF alone was $0.56 per day. The cost of shipping the RUTF supplement from the manufacturer in France to the clinic was approximately $0.43 per day. The total cost of the food basket was approximately $0.68 per day. The cost of transferring each food basket to the clinic was less than $0.01 per day.

**Table 2:** Implementation outcomes, practices, and perceptions of RUTF versus the food basket.

	All subjects n (%)	RUTF n (%)	Food basket n (%)	p-value
**Number of participants, N**	26	12	14	
**Costs**				
Total cost per day, USD		$0.99	$0.68	
Supplement cost, USD		$0.56	$0.68	
Supplement shipping cost, USD		$0.43	< $0.01	
**Fidelity**				
Intervention delivered at every encounter	26 (100.0)	12 (100.0)	14 (100.0)	-
**Uptake**				
Transferred supplement from clinic to household	26 (100.0)	12 (100.0)	14 (100.0)	-
Stored supplement without difficulty	26 (100.0)	12 (100.0)	14 (100.0)	-
Consumed supplement	26 (100.0)	12 (100.0)	14 (100.0)	-
**Acceptability**				
Taste needs improvement	5 (19.2)	**5 (41.7)**	0 (0)	**0.01**
Quantity should be increased	15 (57.7)	1 (8.3)	**14 (100.0)**	**<0.01**
Prefer the other supplement	5 (19.2)	**5 (41.7)**	0 (0)	**0.01**
**Practices**				
Shared supplement	19 (73.1)	5 (41.7)	**14 (100.0)**	**<0.01**
Hid supplement	7 (26.9)	5 (41.7)	2 (14.3)	0.19
Used transportation money to buy food	14 (53.8)	8 (66.7)	6 (42.9)	0.23
**Perceptions of intervention**				
Found supplement useful	26 (100.0)	12 (100.0)	14 (100.0)	-
Improved strength/health	13 (50.0)	7 (58.3)	6 (42.9)	0.43
Improved nutrition / helped with problems obtaining food	15 (57.7)	5 (41.7)	10 (71.4)	0.13
Helped adherence	7 (26.9)	1 (8.3)	6 (42.9)	0.08
Helped household	9 (34.6)	0 (0)	**9 (64.3)**	**<0.01**
**Perceptions of ending the intervention**				
Fear of not having enough food	11 (42.3)	5 (41.7)	6 (42.9)	0.95
Fear of decreased adherence	8 (30.8)	3 (25.0)	5 (35.7)	0.68

### Fidelity and uptake

For both the RUTF and the food basket, the supplement was delivered to 100% of subjects at every study encounter. All subjects reported that they transferred the supplement from the clinic to their household. One subject who received the food basket and one who received RUTF reported that the supplement was “heavy” but not a barrier to transportation. There were no reported problems storing the supplement at home. For both the RUTF and the food basket, the supplement was reported consumed by 100% of subjects.

### Acceptability

Among those who received the RUTF, 5 (42%) suggested that the taste be improved. Specifically, 2 subjects reported that the RUTF was too salty, 1 reported that it was too sweet, and 2 reported that it was too salty and too sweet. All subjects who received the food basket suggested that the quantity be increased and one subject reported that they would have preferred money. Among those who received RUTF, 5 (42%) reported that they would have preferred the food basket; none of those who received the food basket reported that they would have preferred RUTF (p = 0.01).

### Practices

The supplement was shared with household members by 9 (42%) of those who received RUTF versus 14 (100%) of those received the food basket (p<0.01). Among those who received RUTF, 5 (42%) hid the supplement from household members versus 2 (14%) of those who received the food basket (p = 0.19). The majority (75%) of those who received the RUTF consumed it as a broth. Two thirds of those who received RUTF used their transportation money to purchase food compared to 43% of those who received the food basket.

### Perceptions

The supplement was perceived as useful by 100% of subjects. Half of subjects reported that the supplement improved their health and/or strength. A greater percentage of those who received the food basket reported that it improved their nutrition or helped them with their problems obtaining food, that it helped them with adherence, and that it helped the household, compared with those who received RUTF. Individuals in both groups reported that they were afraid they would not have enough food after the study ended, and approximately a third of subjects feared they would have decreased ART adherence.

### Clinical outcomes, nutritional status, and food security

Sputum smear was performed for 23 subjects at enrollment, of which 83% were positive for AFB **(Table 3)**. Sputum smear was performed for all 26 subjects at treatment completion, of which 100% were negative for AFB. For 24 subjects, treatment completion occurred at month 6 and for 2 subjects treatment completion occurred at month 8. Between enrollment and month 6, the median CD4 increased from 207 (IQR 127–313) to 321 (IQR 144–419) and the median hemoglobin increased from 10.2 to 12.8. Indicators of nutritional status included weight, BMI, and percent malnourished. The median weight increased from 50kg at enrollment to 55kg at month 6 and the median BMI increased from 17.3 to 19.3. At enrollment, 58% of subjects were malnourished versus 35% at month 6. Household food security status improved over the course of the study period. The majority of participants (92%) were food insecure at enrollment, and 12 (46%) were severely food insecure. At month 6, 19 (73%) were food insecure and 5 (19%) were severely food insecure. There was no significant difference in clinical outcomes, nutritional status, or food security between those who received RUTF versus the food basket, however the study was not powered to detect outcome differences between assignment groups.

**Table 3:** Clinical outcomes, nutritional status, and food insecurity among participants who received RUTF versus the food basket.

	All subjects	RUTF	Food basket	p-value
**Sputum smear positive**^a^**, n (%)**				
M0	19 (73.1)	10 (83.3)	9 (64.3)	0.54
M6	0	0	0	-
**CD4, median (IQR)**				
M0	207 (127–313)	242 (130–403)	195 (107–285)	0.70
M6	321 (144–419)	326 (216–487)	266 (125–402)	0.66
**Hb (g/dl), median (IQR)**				
M0	10.15 (8.00–11.95)	8.45 (7.90–11.70)	10.50 (8.98–12.18)	0.24
M6	12.75 (11.73–14.00)	11.55 (10.68–12.98)	13.55 (12.55–14.38)	0.18
**Weight (kg), median (IQR)**				
M0	50.00 (44.50–55.00)	47.00 (42.25–55.00)	50.50 (45.00–55.00)	0.45
M6	55.00 (47.00–61.38)	54.50 (45.50–61.63)	55.50 (50.00–62.75)	0.71
**BMI, median (IQR)**				
M0	17.33 (15.98–20.20)	16.76 (15.71–20.20)	18.31 (15.98–19.88)	0.70
M6	19.26 (17.44–23.01)	19.26 (18.11–22.54)	19.12 (17.14–23.39)	1.00
**Malnourished, n (%)**				
M0	15 (57.7)	8 (66.7)	7 (50.0)	0.39
M6	9 (34.6)	4 (33.3)	5 (35.7)	1.00
**Food insecure, n (%)**				
M0	24 (92.3)	11 (91.7)	13 (92.9)	1.00
M6	19 (73.1)	8 (66.7)	11 (78.6)	0.67
**Severely food insecure, n (%)**				
M0	12 (46.2)	7 (58.3)	5 (35.7)	0.25
M6	5 (19.2)	2 (16.7)	3 (21.4)	1.00

### Adherence

Overall 7 day adherence, 4 week adherence, and medication possession exceeded 95% for both ART and TB treatment **(Table 4)**. Adherence did not differ between those who received RUTF and those who received food baskets.

**Table 4:** Overall adherence to ART and TB treatment during the 6 month study period among participants who received RUTF versus the food basket.

	All subjects	RUTF	Food basket	p-value
**Antiretroviral therapy**				
7 day adherence	97.6%	98.6%	96.7%	0.49
4 week adherence	98.8%	98.4%	99.2%	0.59
Medication Possession	99.4%	99.4%	99.4%	0.98
**Tuberculosis treatment**				
7 day adherence	95.9%	96.8%	95.1%	0.62
4 week adherence	98.2%	98.0%	98.4%	0.84
Medication Possession	98.9%	98.5%	99.2%	0.33

## Discussion

We evaluated two different forms of nutrition support, a food basket composed of local food items consistent with local dietary practices and an imported peanut-based RUTF, among a limited sample of HIV-TB co-infected individuals receiving both ART and treatment for TB in Senegal, West Africa. To our knowledge, this is the first study to evaluate the implementation of nutrition support as a strategy to improve ART and TB treatment adherence, TB treatment completion, and clinical outcomes among HIV-TB co-infected individuals in sub-Saharan Africa.

We found that the implementation of both forms of nutrition support for HIV-TB co-infected adults in Senegal is feasible. For both the food basket and the RUTF, the supplement was obtained at every study encounter, transported to the household by the participant, stored, and consumed. However, there were important differences in costs, acceptability, and perceptions of food baskets versus RUTF.

The total cost of both forms of nutrition support, including the cost of transportation, was less than 1 USD per day. Due to differences in transportation costs, the RUTF cost more than the food basket. The food baskets were assembled at the local market and transferred approximately 1 kilometer to the study clinic by car. The RUTF was shipped by boat from the manufacturer in France to Dakar, and then by ferry to the port in Ziguinchor, where it was then transferred by motorized wagon to the study clinic. This difference in transportation costs supports the use of local food baskets over the imported RUTF. Utilization of a local food basket has the additional benefit of supporting the local economy, including support of vendors and farmers in the region.

Sharing of the supplement with the household was more common among those who received the food basket. Although both forms of nutrition support were perceived as useful by participants, a greater proportion of those who received the food basket reported that it helped the household. The finding that all of the participants who received the food basket suggested increasing the quantity of the supplement, likely reflects this frequency of sharing with household members. A greater percentage of individuals who received RUTF hid the supplement from household members. This difference may be partially attributable to the perception that RUTF is a medicine rather than a food product [18], while items in the food basket are part of the traditional diet and require preparation. These differences in practices and perceptions of RUTF versus food baskets are important factors to be considered by programs that are deliberately targeting interventions towards individuals versus households.

Among those who received RUTF, one third reported that the taste of the RUTF needed improvement. Interestingly, although one of the promoted advantages of RUTF is that it does not require preparation, the majority of individuals who received RUTF used it to make broth. Importantly, nearly half of those who received RUTF would have preferred the local food basket. Together, these findings suggest that the local food basket may be more acceptable, at both the individual and household level, in this population.

All outcomes, including CD4 cell count, hemoglobin, nutritional status, and food security, improved over the study period and adherence to TB treatment and ART exceeded 95% by all adherence measures utilized. Although a greater proportion of those who received food baskets reported that the supplement helped them with adherence, we did not find a measurable difference in adherence or treatment completion between groups. All subjects completed TB treatment and were smear negative at treatment completion. This represents a substantial improvement compared to the treatment success rate of 54% reported among HIV-positive TB cases nationally [5].

Prior studies to evaluate macronutrient nutrition support for TB infected individuals in Sub-Saharan Africa are minimal, and none have evaluated ART and TB medication adherence among individuals co-infected with TB and HIV [9, 11]. In Tanzania, supplementation in the form of biscuits has been evaluated in two related studies [19, 20]. The first study compared weight gain and changes in body composition among 865 TB-infected individuals receiving 2 months of supplementation with a micronutrient fortified biscuit versus supplementation with a non-fortified biscuit [19]. Approximately 28% of subjects in the study were HIV-positive, 22% of whom were receiving ART during the study. There was no difference in weight gain at 8 or 20 weeks. TB and HIV outcomes, including adherence, were not monitored. A follow-up study in Tanzania was conducted to evaluate rifampin pharmacokinetics among those receiving the fortified biscuit for 2 months [20]. Among 100 TB-infected subjects, 50% were HIV-infected and none were receiving ART. Although not the focus of the study, adherence to TB treatment was reported. Among those receiving the biscuits, 2 individuals (3.9%) had <95% adherence versus 6 individuals (12.3%) who were not receiving the biscuits. Methods for determining adherence were not described.

In a study among 100 TB-infected, HIV-negative individuals in India, provision of a locally made ready-to-eat food composed of wheat flour and groundnuts for 3 months was associated with greater weight gain, and higher rates of treatment completion and sputum conversion compared to controls [21]. In a subsequent study in India, a monthly ration of a powdered cereal-lentil mixture was provided to 103 TB-infected adults for 6 months [22]. Approximately 21% of subjects were HIV-infected and none were receiving ART. There was no difference in weight gain, tuberculosis treatment outcomes, or HIV outcomes among those who received the mixture compared to controls. Among 265 TB-infected, HIV-negative individuals in Timor Leste, the provision of a daily meal resulted in greater weight gain at 8 and 32 weeks compared to controls [23]. There was no difference in treatment completion or sputum conversion. In Singapore, high-energy nutritional supplementation to 36 TB-infected, HIV-negative individuals resulted in greater weight gain at 6 weeks compared to controls [24]. Tuberculosis treatment outcomes were not compared. In a retrospective comparison of two groups of TB patients in Brazil of which 4.3% were HIV-positive, the group that received a food basket (N = 68) had higher cure and treatment completion rates compared to those who received TB treatment alone (N = 74) [25].

Importantly, the majority of subjects in our study remained food insecure at study completion, and subjects commonly reported that they feared they would not have enough food after the study ended. Although household food insecurity scores improved over the course of the study period, these improvements may solely be a reflection of receipt of the study intervention, in which case they would be expected to be transient. Alternatively, they may be indicative of progressive clinical improvement resulting from successful treatment of active TB and HIV, which leads to decreased household medical expenditures and enables subjects to seek employment.

While lifelong nutrition support may not be a feasible strategy, our findings suggest that temporary support during the critical months of treatment against active TB, could contribute to improved adherence and treatment completion, and subsequently, improved clinical and socioeconomic outcomes. Importantly, sharing of both forms of nutrition support with household members was frequently reported in this study and is consistent with cultural norms and findings from previous studies [18, 26, 27]. Knowledge of household dietary practices and consideration and calculation for household sharing is essential in order for nutrition interventions to be effective.

The primary limitation of this study was sample size. This was a pilot implementation study and it was not powered to detect differences in clinical outcomes between groups. Viral load monitoring was not available at the study sites during the study period, therefore we were unable to capture rates of virologic failure. Additionally, we did not compare clinical outcomes among those who received nutrition support to those who did not receive any form of nutrition support, as a control group was not included due to ethical considerations.

## Conclusion

Our findings suggest that the implementation of nutrition support for HIV-TB co-infected adults in Senegal is feasible and may provide an effective strategy to improve adherence, treatment completion, and clinical outcomes for less than 1 USD per day. Further studies to determine the impact of nutrition support among a larger population of HIV-TB co-infected individuals are indicated.

## Supporting information

S1 Fig CONSORT flow diagram. (TIFF)

S1 File CONSORT checklist. (PDF)

S2 File Protocol. (PDF)
