# Supplementary figures and images for: Nutrition support for HIV-TB co-infected adults in Senegal, West Africa: A randomized pilot implementation study
Source: PLoS One. 2019 Jul 18;14(7):e0219118. doi: 10.1371/journal.pone.0219118 (PMC6638996; doi:10.1371/journal.pone.0219118)

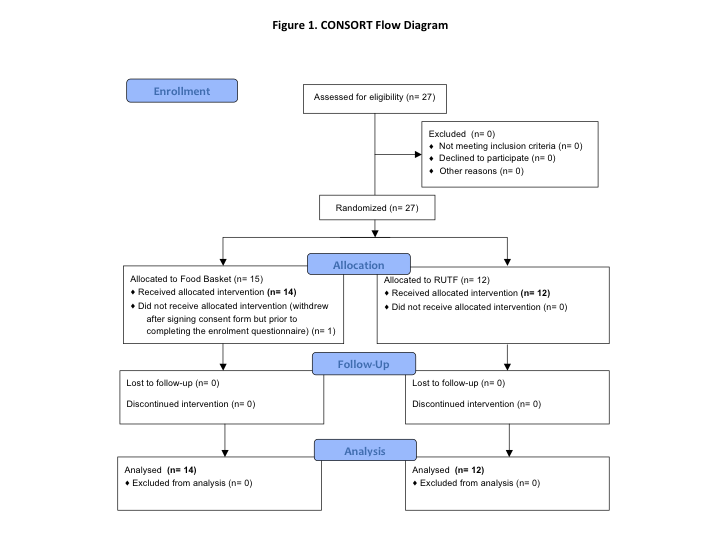

Supplement: S1 Fig — (TIFF) [file pone.0219118.s001.tiff]
