# Supplementary material for: Nutrition support for HIV-TB co-infected adults in Senegal, West Africa: A randomized pilot implementation study
Source: PLoS One. 2019 Jul 18;14(7):e0219118. doi: 10.1371/journal.pone.0219118 (PMC6638996; doi:10.1371/journal.pone.0219118)
Supplement: S2 File — (PDF) [file pone.0219118.s003.pdf]

UNIVERSITY OF WASHINGTON

Human Subjects Division
Box 359470
APPLICATION: Human Subjects Review

BOX FOR COMMITTEE USE ONLY		
MASTER O	COMM. O	INVESTIGATOR O
APPLICATION NO.	50147-J	

RECEIVED
Human Subjects Division
JUL 20 2015
UW

I. PRINCIPAL INVESTIGATOR (Provide all the information requested. Change of PI requires a modification. All paper-based correspondence will be directed to this person. Please list the mailing address for paper-based correspondence. You may designate a contact person other than yourself in section II., below.)

Name Noelle A. Benzekri Title M.D. Position Fellow

Home Institution (or source of paycheck) UW
UW Student? Home institution is UW.

Home UW Department (if applicable) Medicine Division Allergy and Infectious Diseases

UW Position or appointment (choose the most appropriate one):

Faculty:
[ ] Regular Faculty Appointment [ ] Research Faculty Appointment [ ] Clinical Faculty Appointment
[ ] Visiting Faculty Appointment [ ] Dual Appointment with PNNL
[ ] Other (describe):

Student:
[ ] Matriculated Undergraduate Student [ ] Graduate or Professional Student (matriculated or approved "On Leave") [ ] WWAMI Student

[X] Resident or Fellow at the UW or Local VA [ ] UW Administration or Staff [ ] None

Campus Box # 358061 Other Address if not at UW

Telephone 206-543-9842 Fax 206-616-4898 e-mail benzekri@uw.edu

II. IRB CONTACT PERSON (Provide all the information requested. Change of Contact Person requires a modification. If this section is completed, all paper-based correspondence will be directed to this person.)

Name Title Position

Home Institution (or source of paycheck)

Home UW Department (if applicable) Division

UW Position or appointment (choose the most appropriate one):

Faculty:
[ ] Regular Faculty Appointment [ ] Research Faculty Appointment [ ] Clinical Faculty Appointment
[ ] Visiting Faculty Appointment [ ] Dual Appointment with PNNL
[ ] Other (describe):

Student:
[ ] Matriculated Undergraduate Student [ ] Graduate or Professional Student (matriculated or approved "On Leave") [ ] WWAMI Student

[ ] Resident or Fellow at the UW or Local VA [ ] UW Administration or Staff [ ] None

Campus Box # Other Address if not at UW

Telephone Fax e-mail

III. TITLE OF PROJECT:
A pilot study of nutritional supplementation for malnourished HIV-TB co-infected adults in Senegal

IV. SIGNATURES: The undersigned acknowledge that: 1. this application is an accurate and complete description of the proposed research; 2. the research will be conducted in compliance with the recommendations of and only after approval has been received from the Institutional Review Board (IRB). The lead researcher is responsible for all aspects of this research, including: reporting any serious adverse events or problems to the IRB, requesting prior IRB approval for modifications, and requesting continuing review and approval.

A. Investigator: Noelle A. Benzekri [signature] 7/16/15
TYPED NAME PLUS SIGNATURE DATE

B. Faculty sponsor (for student): Change requires a modification. Geoffrey S. Gottlieb [signature] 7/16/15
TYPED NAME PLUS SIGNATURE DATE

C. The Chair, Dean, or Director acknowledges the researcher is qualified to do the research, sufficient resources will be available, and (if no external funding review occurred) there was an internal review of scientific merit.
Wesley Van Voorhis [signature] 7/16/15
TYPED NAME PLUS SIGNATURE DATE

V. TYPE OF NEW SUBMISSION

[ ] **IRBshare**

[ ] **MINIMAL RISK**
(The research meets the **definition of minimal risk** and falls into one or more **expedited review categories**. **You are done with this table.**)

[X] **FULL COMMITTEE**

- [X] Full Committee (The research involves **greater than minimal risk** and requires review at a convened meeting of the IRB)
- [ ] Full Committee (The research involves **no more than minimal risk** but does not fit into one of the expedited review categories)

[X] **METHODS:**

Please mark the boxes to indicate the methods which best describe your study:

[ ] **Social-Behavioral Procedures/Considerations**

[ ]	Observational	[ ]	Population-Based Field Study
[ ]	Behavioral Interventions	[X]	Surveys/Questionnaires
[X]	Interview/Focus Groups	[ ]	Other – Describe:

[ ] **Medical Procedures/Considerations**

[ ]	Bio-hazardous Substances	[ ]	Investigational/Approved Devices
[ ]	Controlled Substances	[ ]	Radiation Exposure
[ ]	Emergency Treatment	[ ]	Substance Abuse Treatment (with medication)
[ ]	Gene Transfer Study	[ ]	Surgical Procedures
[ ]	Stem Cell Research	[ ]	Genetic Testing
[ ]	Magnetic Resonance imaging (MRI)	[ ]	Complementary/Alternative Medicine
[ ]	Investigational/Approved Drugs and Biologics	[X]	Other – Describe: Nutritional supplementation

[ ] **DOES YOUR RESEARCH INVOLVE OR IS IT ASSOCIATED WITH ANY OF THE FOLLOWING:**

- [ ] Emergency Medicine
- [ ] Pregnant Women as a Target Population
- [ ] Genetics
- [ ] Stem Cells
- [ ] Neuroscience
- [ ] College of Arts and Sciences
- [ ] College of Education
- [ ] Dentistry
- [X] Infectious Disease
- [X] HIV/AIDS
- [ ] Psychiatry
- [ ] Rehabilitation Medicine
- [ ] Psycho-Social Drug Abuse Research
- [ ] Alaska Native/American Indian (ANAI)
- [X] Public Health
- [X] Global Health
- [X] Health Services
- [X] Quality of Care / Quality of Life
- [ ] Health Prevention / Health Education
- [ ] Nursing

[X] Confirm by checking this box that you are listing on Section VIII all direct and indirect funding that supports this research. Indirect funding is generic (i.e., not tied to this specific study) federal salary support for the time that any key personnel spend on the research. Examples: many training grants, fellowships, scholarships, and career development awards.

VI. PRIMARY RESEARCH ROLES

Some research projects are conducted by a large team of individuals. Other projects can be performed by only one or two individuals. The IRB does not need to know the name of every member of your research team - instead, the IRB wants to know who is fulfilling the following specific roles for your research. Note that the same individual may play multiple roles. If it is necessary to identify an individual by name, this will be specified below. Each section below must be completed.

1. Information for **individuals identified by name:**

The individuals below need to be identified by name. If these individuals change during the course of the research, a Modification approval from the IRB is needed before making the change.

Subject Contact Person (to answer questions, receive complaints or reports of side effects, etc.)

Check here if the same as: [ ] Lead Researcher [ ] IRB Contact Person
If one of these boxes is checked, you do not need to complete the rest of this table.

Name Ibrahima Sall Title ____ Position Ziguinchor Study Coordinator

Home Institution (or source of paycheck)
UW Student? Home institution is UW. Centre de Santé de Ziguinchor, Senegal

Home UW Department (if applicable) ____ Division ____

UW Position or appointment (choose the most appropriate one):

Faculty: [ ] Regular Faculty Appointment [ ] Research Faculty Appointment [ ] Clinical Faculty Appointment
[ ] Visiting Faculty Appointment [ ] Dual Appointment with PNNL
[ ] Other (describe): ____

Student: [ ] Matriculated Undergraduate Student [ ] Graduate or Professional Student (matriculated or approved "On Leave") [ ] WWAMI Student

[ ] Resident or Fellow at the UW or Local VA [ ] UW Administration or Staff [ ] None

Campus Box # ____ Other Address if not at UW Centre de Santé de Ziguinchor, Ziguinchor, Senegal

Telephone 77-645-3779 Fax ____ e-mail ibasall961@gmail.com

Study Coordinator

Check here if the same as: [ ] Lead Researcher [ ] IRB Contact Person [X] Subject Contact Person
If one of these boxes is checked, you do not need to complete the rest of this table.

Name ____ Title ____ Position ____

Home Institution (or source of paycheck)
UW Student? Home institution is UW. ____

Home UW Department (if applicable) ____ Division ____

UW Position or appointment (choose the most appropriate one):

Faculty: [ ] Regular Faculty Appointment [ ] Research Faculty Appointment [ ] Clinical Faculty Appointment
[ ] Visiting Faculty Appointment [ ] Dual Appointment with PNNL
[ ] Other (describe): ____

Student: [ ] Matriculated Undergraduate Student [ ] Graduate or Professional Student (matriculated or approved "On Leave") [ ] WWAMI Student

[ ] Resident or Fellow at the UW or Local VA [ ] UW Administration or Staff [ ] None

Campus Box # ____ Other Address if not at UW ____

Telephone ____ Fax ____ e-mail ____

2. Information for **research staff who will perform procedures that involve risk to subjects:**

The individuals below do not need to be identified by name, rather, by qualifications. As long as the qualifications of the individuals and the procedures performed remain the same, a modification is not needed.

If an individual is **not** an agent of the UW, indicate his/her institution or organization. Should an individual not be associated with an institution or organization, state so. For all non-UW individuals, it will be necessary for this individual to receive IRB review. There are a number of mechanisms by which this may occur.

- If the non-UW individual is associated with Pacific Northwest National Laboratories, Puget Sound Blood Center, King County-Seattle Public Health, or Northwest Kidney Center: the UW has an institutional **Authorization Agreement** with these institutions by which it provides the IRB review (as long as any funding is administered through the UW).

- If the non-UW individual is associated with the Fred Hutchinson Cancer Research Center, Group Health, Seattle children's, Swedish Medical Center, or Benaroya Research Institute at Virginia Mason: the terms of the UW's **Cooperative IRB Agreement** with these institutions may or may not allow the UW IRB to do the review. The individual should contact their institution's IRB for guidance.
- If the non-UW individual is associated with an institution or organization in which the UW **does not** have a Cooperative IRB Agreement, it will be necessary for the non-UW individual to provide their own IRB review. If the non-UW individual's institution or organization does not have their own IRB or does not use an IRB for review of their research and the non-UW individual's institution or organization has a Federalwide Assurance (FWA), the non-UW individual's institution or organization may enter into an **IRB Authorization Agreement** with the UW. This means that the UW will provide IRB review for the non-UW individual. The non-UW Individual's institution or organization may also wish to enter into an IRB Authorization Agreement even if they have their own IRB to prevent duplication of effort. However, entering into an IRB Authorization Agreement with a non-UW individual's institution or organization is at the discretion of the HSD.
- If the non-UW individual is not associated with an institution or organization or if the non-UW individual is associated with an institution or organization that does not have a FWA and does not routinely conduct research, an **Individual Investigator Agreement** may be entered into with the UW. The Individual Investigator Agreement extends the applicability of the UW's FWA to cover the non-UW individual, institution or organization. However, entering into an Individual Investigator Agreement with a non-UW individual, institution or organization is at the discretion of the HSD.

Please see SOP Authorization Agreements for information on how to obtain an Agreement for your research.

Study Procedures that involve risk to subjects

[X] Phlebotomy (blood draw)

Who will perform this procedure?

[X] Licensed Practitioner

Describe the qualifications for the Licensed Practitioner below, including whether they are affiliated with the UW, any requirement for professional license or credential, and/or experience in performing this procedure:

This will be a licensed practitioner authorized by the Centre de Santé de Ziguinchor to perform phlebotomy for clinic patients and trained in universal precautions procedures.

[ ] Study Nurse

Describe the qualifications for the Study Nurse below, including whether they are affiliated with the UW, any requirement for professional license or credential, and/or experience in performing this procedure:

[ ] Other

Describe the qualifications for the Other Professional below, including whether they are affiliated with the UW, any requirement for professional license or credential, and/or experience in performing this procedure:

[ ] MRI Scan

Who will perform this procedure?

[ ] Licensed Practitioner

Describe the qualifications for the Licensed Practitioner below, including whether they are affiliated with the UW, any requirement for professional license or credential, and/or experience in performing this procedure:

[ ] Study Nurse

Describe the qualifications for the Study Nurse below, including whether they are affiliated with the UW, any requirement for professional license or credential, and/or experience in performing this procedure:

[ ] Other:

Describe the qualifications for the Other Professional below, including whether they are affiliated with the UW, any requirement for professional license or credential, and/or experience in performing this procedure:

[ ] Surgical or Physically Invasive Procedure

Who will perform this procedure?

[ ] Licensed Practitioner

Describe the qualifications for the Licensed Practitioner below, including whether they are affiliated with the UW, any requirement for professional license or credential, and/or experience in performing this procedure:

[ ] Study Nurse

Describe the qualifications for the Study Nurse below, including whether they are affiliated with the UW, any requirement for professional license or credential, and/or experience in performing this procedure:

Describe the qualifications for the Other Professional below, including whether they are affiliated with the UW, any requirement for professional license or credential, and/or experience in performing this procedure:

[X] Other Procedures Involving Risk to Subjects [name the procedure(s)]: Interpretation of physical exam and/or lab results
Examples: behavioral therapy; dietary counseling; assessments and/or interpretations of test reults that require specific expertise (e.g. physical exam; fitness assessment; cognitive state; suicidality; mental health; interpretation of imaging tests, genetic tests, cognitive tests, etc.) *For more than one "Other" procedures, copy and paste this portion of the table as many times as necessary.*

Who will perform this procedure?

[X] Licensed Practitioner

Describe the qualifications of the Licensed Practitioner below, including whether they are affiliated with the UW, what is their professional license, and their experience in performing this procedure:

Clinic physician with experience providing HIV and TB care.

[ ] Study Nurse

Describe the qualifications of the Study Nurse below, including whether they are affiliated with the UW, what is their professional license, and their experience in performing this procedure:

[ ] Other:

Describe the qualifications of the Other Professional below, including whether they are affiliated with the UW, what is their professional license, and their experience in performing this procedure:

[ ] Other Procedures Involving Risk to Subjects [name the procedure(s)]: Interviews and questionnaires
Examples: behavioral therapy; dietary counseling; assessments and/or interpretations of test reults that require specific expertise (e.g. physical exam; fitness assessment; cognitive state; suicidality; mental health; interpretation of imaging tests, genetic tests, cognitive tests, etc.) *For more than one "Other" procedures, copy and paste this portion of the table as many times as necessary.*

Who will perform this procedure?

[X] Licensed Practitioner

Describe the qualifications of the Licensed Practitioner below, including whether they are affiliated with the UW, what is their professional license, and their experience in performing this procedure:

Clinic social worker with experience providing care to HIV and TB infected patients.

[ ] Study Nurse

Describe the qualifications of the Study Nurse below, including whether they are affiliated with the UW, what is their professional license, and their experience in performing this procedure:

[ ] Other:

Describe the qualifications of the Other Professional below, including whether they are affiliated with the UW, what is their professional license, and their experience in performing this procedure:

3.a. **Non-UW Individuals, Institutions or Organizations.** It will be necessary for each non-UW individual, institution or organization listed below to receive IRB review of its involvement in this research. There are a number of mechanisms by which this may occur.

Please only list the non-UW individual, institution or organization below if you are:

- **The direct recipient of an award or if you will be providing funding to the non-UW individual, institution or organization through a mechanism such as a sub-contract; and**
- **If the non-UW individual, institution or organization will be acting on behalf of the UW research study to do any of the following: 1) Obtain consent from subjects, 2) Perform procedures involving subject interaction or observation, 3) Obtain identifiable data/specimens, 4) Have access to, or receive coded or identifiable data/specimens, 5) Intervene by manipulating the environment.**
- If the non-UW individual is associated with Pacific Northwest National Laboratories, Puget Sound Blood Center, King County-Seattle Public Health, or Northwest Kidney Center: the UW has an institutional Authorization Agreement with these institutions by which it provides the IRB review (as long as any funding is administered through the UW).

- If the non-UW individual is associated with the Fred Hutchinson Cancer Research Center, Group Health, Seattle children's, Swedish Medical Center, or Benaroya Research Institute at Virginia Mason: the terms of the UW's **Cooperative IRB Agreement** with these institutions may or may not allow the UW IRB to do the review. The individual should contact their institution's IRB for guidance.
- If the non-UW individual, institution or organization is one in which the UW **does not** have a Cooperative IRB Agreement, it will be necessary for the non-UW individual, institution or organization to provide their own IRB review. If the non-UW individual, institution or organization does not have their own IRB or does not use an IRB for review of their research, and the non-UW individual, institution or organization has a Federal Wide Assurance (FWA), the non-UW individual, institution or organization may enter into an **IRB Authorization Agreement** with the UW. This means that the UW will provide IRB review for the non-UW individual, institution or organization. The non-UW individual, institution or organization may also wish to enter into an IRB authorization Agreement even if they have their own IRB to prevent duplication of effort. However, entering into an IRB Authorization Agreement with a non-UW individual, institution or organization is at the discretion of the HSD.
- If the non-UW individual is not associated with an institution or organization that has a Federal Wide Assurance (FWA) or if the institution or organization listed below does not have a FWA and does not routinely conduct research, an **Individual Investigator Agreement** may be entered into with the UW. The Individual Investigator Agreement extends the applicability of the UW's FWA to cover the non-UW individual, institution or organization. However, entering into an Individual Investigator Agreement with a non-UW individual, institution or organization is at the discretion of the HSD.

Please see SOP Authorization Agreements for information on how to obtain an Agreement for your research.

Additional IRB approval will be obtained from the Senegal Comité National d'Ethique pour la Recherche en Santé prior to commencing research.

3b. Non-UW Individual, Organization or Location:

If there is more than one non-UW individual, organization, or location, copy and paste this table as many times as necessary.

Name of the non-UW Individual, Organization or Location:

Centre de Santé de Ziguinchor, Ziguinchor, Senegal

Address of the non-UW Individual, Organization or Location:

Centre de Santé de Ziguinchor, Ziguinchor, Senegal

Describe the activities that will be performed by/at the non-UW Individual, Organization or Location (if the specified activities will not be performed, please enter N/A):

Obtain consent from the subjects:	Informed consent will take place at the Centre de Santé de Ziguinchor.
Perform procedures involving subject interaction or observation:	Study interactions will take place at the Centre de Santé de Ziguinchor.
Obtain identifiable data/specimens?	Identifiable data will be obtained and stored at the Centre de Santé de Ziguinchor.
Have access to, or receive coded or identifiable data/specimens:	Study staff will have access to identifiable data (ex. medical records, lab specimens, pharmacy records) at the Centre de Santé de Ziguinchor.
Intervene by manipulating the environment:	Nutritional supplementation will be distributed at the Centre de Santé de Ziguinchor.

Name of the non-UW Individual, Organization or Location:

Centre Hospitalier Universitaire de Fann, Université Cheikh Anta Diop de Dakar, Senegal

Address of the non-UW Individual, Organization or Location:

Ibrahima DIOP MAR - Centre Hospitalier Universitaire de Fann, BP 5035, Dakar, Senegal

Describe the activities that will be performed by/at the non-UW Individual, Organization or Location (if the specified activities will not be performed, please enter N/A):

Obtain consent from the subjects:	N/A

Perform procedures involving subject interaction or observation:	N/A
Obtain identifiable data/specimens?	N/A
Have access to, or receive coded or identifiable data/specimens:	Study staff will have access to identifiable data/lab specimens
Intervene by manipulating the environment:	N/A

VII. SECTION 1 - LIST EACH PROPOSED AND FUNDED GRANT OR CONTRACT RELEVANT TO THIS APPLICATION, AND ATTACH A COMPLETE COPY OF EACH GRANT OR CONTRACT. THIS SHOULD INCLUDE GRANTS THAT PROVIDE GENERAL SALARY SUPPORT TIME SPENT ON THE RESEARCH (E.G. A TRANING GRANT OR CAREER DEVELOPMENT AWARD). IF NONE, CHECK HERE ☐. FOR CENTER OR PROGRAM PROJECT GRANTS LIST P.I. AND TITLE FOR EACH SEPARATE PROJECT OR CORE. ADD SHEETS IF NECESSARY.

For Center, Program, and Institutional Training Grants (e.g., NIH "P" awards and "T" awards): Attach only the following components of the application. The terms used here are from the standard NIH applications for these types of grants. If the grant is from another agency, provide the equivalent application sections.

- Cover page(s)
- Project/Performance Site Location
- Other Project Information
- Research Plan (for Center or Program grants)
- Research Training Program Plan (for Institutional Training grants)
- Biosketch (profile) for the principal investigator on the grant

For Department of Defense (DOD) funding, complete and attach the SUPPLEMENT: Department of Defense
For Department of Justice (DOJ) funding, complete and attach the SUPPLEMENT: Department of Justice

A. Type of proposal: ☒ Research ☐ Contract ☐ Fellowship ☐ Training grant ☐ Subcontract
☐ Other, specify

B. Name of principal investigator: Noelle A. Benzekri

C. Name of funding agency: The Firland Foundation

D. Agency's number (if assigned):

E. Title of proposal: A pilot study of nutritional supplementation for malnourished HIV-TB co-infected adults in Senegal

F. Inclusive dates: from 9/1/2015 through 9/1/2017

G. Status: ☒ New ☐ Competing renewal ☐ Non-competing renewal

H. Submitted through UW Office of Sponsored Programs? ☒ Yes ☐ No, (attach explanation)

VIII. SUMMARY OF ACTIVITY. Answer in spaces provided (add numbered, referenced, single-sided sheets when necessary). Do not refer to an accompanying grant or contract proposal.

A. BACKGROUND AND PURPOSE OF RESEARCH. Provide relevant background information and explain **in lay language** why this research is important and what question(s) or hypotheses this activity is designed to answer.

Malnutrition is associated with increased mortality and worse clinical outcomes among individuals infected with HIV and tuberculosis (TB). However, the advantages of nutritional supplementation on clinical outcomes among HIV-TB co-infected individuals in regions with high rates of food insecurity is unknown. Studies evaluating the potential benefits of nutritional supplementation are urgently needed to inform national HIV and tuberculosis treatment programs. This is particularly true in regions with high rates of food insecurity, such as Senegal, West Africa. Based on our interviews conducted with HIV infected adults in Senegal, the majority (87%) suffer from food insecurity. Based on our previous studies conducted in Senegal from 1994-2010, the prevalence of malnutrition (Body Mass Index <18.5) among 1213 HIV positive adults was 44%. TB is the leading cause of hospitalization and death among HIV infected adults in Senegal[11] and is a major contributor to malnutrition and wasting.

The proposed pilot study will take place at our established study site at the Centre de Santé de Ziguinchor located in the Casamance region of Senegal. This region has the highest prevalence of HIV in the country, in addition to the highest rates of food insecurity and [illegible]

HIV-TB co-infected adults initiating treatment for tuberculosis. The results of this study will then be used to design and seek funding to conduct a larger randomized clinical trial of nutritional supplementation to improve clinical outcomes, including increased completion of treatment for tuberculosis, among HIV-TB co-infected individuals in Senegal.

Aim 1. Determine the acceptability and impact on weight gain of nutritional supplementation with ready-to-use therapeutic food (RUTF) versus monthly food rations among malnourished HIV-TB co-infected adults.
Hypothesis: Individuals will prefer monthly food rations over RUTF. However, monthly food rations will be shared with household members, resulting in less weight gain among those who receive monthly food rations compared to those who receive RUTF.

Aim 2. Compare different methodologies for measuring medication adherence. Determine rates of medication adherence and rates of completion of treatment for tuberculosis.
Hypothesis: Self reported measures will underestimate medication adherence when compared with the Medication Possession Ratio. Nutritional supplementation will be associated with increased rates of treatment completion when compared with previously reported rates of treatment completion in Senegal. Medication adherence and treatment completion will be greater among those receiving monthly food rations compared to RUTF.

Aim 3. Determine rates of immunologic recovery and virologic failure.
Hypothesis: Nutritional supplementation will be associated with greater rates of immunologic recovery and decreased rates of virologic failure when compared with previously reported rates in Senegal. Virologic failure rates will be lowest among those receiving food rations compared to RUTF.

B. RESEARCH PROCEDURES INVOLVED.

1. Provide a complete description of: a. the study design, and b. sequence and timing of all study procedures that will be performed, e.g., volume of blood, size of biopsy, drug administration, questionnaire, name of psychological test. Provide this information for each phase of the study (pilot, screening, intervention and follow-up). **Use lay language**. Attach study flow sheet, if available.

Procedure	Pre-screening	Enrollment encounter	Monthly follow-up encounters (months 1-5)	6 month follow-up encounter
Pre-screening of medical records	X			
Screening		X		
Consent		X		
Randomization		X		
Questionnaires/Interviews		X	X	X
Chart review		X	X	X
Height/weight		X	X	X
Mid-Upper Arm Circumference		X	X	X
WHO staging		X		
Blood draw		X		X
Dispense 1 month supply of nutritional supplement		X	X	
Adherence assessment			X	X

This study will be conducted among HIV-TB co-infected adults initiating treatment for tuberculosis. There will be a total of 7 study encounters for each participant, including the enrollment visit. Study encounters will take place monthly for 6 months while the subject is receiving treatment for tuberculosis.

Pre-screening: Clinic charts will be reviewed to identify potentially eligible subjects and clinic staff will be informed of study enrollment criteria. Clinic staff will be asked to refer potentially eligible subjects to the study social worker.

Screening and Consent: Subjects will be referred to a private area for screening and consent. Informed consent will be conducted by the social worker. Subjects will be randomized 1:1 to receive nutritional supplementation with either Plumpy'Nut ready-to-use therapeutic food or a monthly food ration.

Nutritional supplements: Plumpy'Nut ready-to-use therapeutic food is commonly used by international aid organizations during relief efforts. It is a micronutrient fortified peanut paste containing milk-powder and vegetable fat. Those randomized to receive food rations will receive a monthly food basket containing cooking oil, rice, local peas, and powdered milk. Plumpy'Nut provides approximately 1000kcal, 25g protein, and 66g fat per day and the food ration provides approximately 1100kcal, 28g protein, and 65g fat per day.

Participants will receive a 30 day supply of either Plumpy'Nut or food rations each month during their 6 months of treatment for tuberculosis.

Enrollment encounter: Subjects will complete questionnaires and participate in interviews to assess food insecurity, dietary diversity, coping strategies, nutritional and economic behaviors, perceptions of nutritional interventions, and medical and social history. Clinical records will be further reviewed to record details of the subject's medical history. Subject height, weight, and mid-upper arm circumference will be measured and a clinical evaluation will be performed to document WHO clinical stage. Subjects will undergo a 30mL blood draw to measure CD4 and viral load. Subjects will receive a month supply of either Plumpy'Nut or food rations.

Monthly follow-up encounters: Subjects will complete questionnaires and participate in interviews. Subject height, weight, and mid-upper arm circumference will be measured. Adherence will be determined by subject report and review of pharmacy records. Subjects will receive a month supply of either Plumpy'Nut or food rations at each encounter during months 1-5. The 6 month follow-up encounter will include a 30mL blood draw to measure CD4 and viral load.

2. Would subjects undergo these or similar procedures (medical, psychological, educational, etc.) if they were not taking part in this research? ☒No ☒ Yes If "Yes," describe how the study procedures differ from what subjects would otherwise undergo.

   Subjects undergo clinical evaluation and blood draw as part of routine care. Study procedures will differ from what subjects would otherwise undergo due to the addition of anthropometric measurements, participation in interviews and questionnaires, assessments of food insecurity and dietary diversity, and the provision of nutritional supplementation. Subjects participating in this study will have an additional 30mL of blood drawn at enrollment and 6 month follow-up to determine CD4 and viral load.

3. Check all of the boxes below that apply to your research:

   Drug administration

   ☐ Administration of a drug (either FDA-approved or investigational) for research purposes to a subject-patient during general or regional anesthesia.

   ☐ Administration of a drug (either FDA-approved or investigational) for research purposes to a subject-patient during the 1.5 hours preceding general or regional anesthesia.

   Blood lines

   ☐ Inserting an intravenous (central or peripheral) or intra-arterial line for research purposes in a subject-patient **during** general or regional anesthesia.

   Sample collection

   ☐ Obtaining samples of blood, urine, or cerebrospinal fluid for research purposes while a subject-patient is under general or regional anesthesia.

   ☐ Obtaining a research sample from tissue or organs that would not otherwise be removed during surgery, while the subject-patient is under general or regional anesthesia.

   Radio-isotopes

   ☐ Administration of a radio-isotope for research purposes during the 3 hours prior to anesthesia or while a subject-patient is under general or regional anesthesia.

   If you checked this box, you are responsible for informing **in advance** all appropriate clinical personnel (e.g., nurses, technicians, anesthesiologists, surgeons) about the administration and use of the radio-isotope, to ensure that any personal safety issues (e.g., pregnancy) can be appropriately addressed. This is a condition of IRB approval.

   Experimental devices

   ☐ Implantation of an experimental device while a subject-patient is under general or regional anesthesia.

   Other experimental manipulations or procedures

   ☐ Other manipulations or procedures performed solely for research purposes while a subject-patient is under general or regional anesthesia (e.g., experimental liver dialysis, experimental brain stimulation)

   None of the above

   ☒ None of the above apply to my research

4. If you checked any box in question #3 except "none of the above", answer the following questions:

a. Provide the name and institutional affiliation of the physician anesthesiologist who is a member of your research team or who will serve as a safety consultant about the interactions between your research procedures and the general or regional anesthesia of the subject-patients. If your procedures will be performed at a UW Medicine facility or affiliate, the anesthesiologist must be a UW faculty member.

b. If you have not yet consulted with an appropriately qualified person about this issue, describe in detail your plans to do so. The IRB will not approve your application without this consultation. If UW Department of Anesthesiology approval has been obtained, please provide the Department's letter of support.

5. Required application supplements. Complete and attach the indicated SUPPLEMENT, as appropriate

a. **SUPPLEMENT: Drugs, Biologics, Botanicals** – for research involving the use of any of the following:

- Drugs regulated by the FDA (prescription, over-the-counter, approved, or investigational)
- Biologics regulated by the FDA (prescription, over-the-counter, approved, or investigational)
- Botanicals
- Dietary Supplements

b. **SUPPLEMENT: Devices** – for research involving the use of any medical device (approved or investigational; including software used with a medical device, and including mobile medical applications).

c. **SUPPLEMENT: Genetic Research** – submit this supplement when your research involves genetics. **Genetic research** is defined as research involving the analysis of any of the following: DNA; RNA; chromosomes; mitochondria; any or all parts of the human genome; or biomarkers such as proteins or metabolites which may be implicated in, associated with, or cosegregated with a disorder, syndrome, condition, or predisposition to disease or behavior. Usually genetic research involves the collection and/or use of human biological specimens such as blood, skin, or other tissues, nail clippings, or hair. Genetic research may also include the construction of pedigrees ("maps" of the distribution of a particular trait or condition among related individuals) or family medical histories.

d. **SUPPLEMENT: Department of Defense** – for research involving any component of the federal Department of Defense (DOD). "Involvement" means funding; collaboration or cooperative arrangements; use of facilities, resources, or personnel; use of military or civilian members of the DOD (or their records/specimens) as subjects.

e. **SUPPLEMENT: Department of Justice** – for research involving the federal Department of Justice (DOJ) or any of its components (such as the National Institute of Justice, or any facilities/personnel of the Bureau of Prisons). "Involvement" means funding; collaboration or cooperative arrangements; use of facilities, resources, or personnel; use of records or specimens from DOJ employees or from prisoners in any Bureau of Prisons facility.

f. **SUPPLEMENT: GWAS dbGaP** – for research that will involve submitting data to the federal Database of Genotyped and Phenotyped (dbGaP) information.

g. For research involving the **Department of Energy (DOE)**, researchers should consult the **CHECKLIST Department of Energy** to ensure that they have addressed all DOE requirements. However, the Checklist does not need to be completed and submitted unless the researcher believes it would be a useful attachment.

C. DECEPTION: If any deception or withholding of complete information is required for this activity, explain why this is necessary and attach a protocol explaining if, how, when, and by whom subjects will be debriefed.

This study does not involve deception or withholding.

D. SUBJECTS

The IRB reviews the number of subjects you plan to study in the context of risks and benefits. If your research is approved for a specific number of subjects, the data from any "extra" subjects cannot be described as having been obtained with IRB approval.

See the HSD website for the definition of "human subject" http://www.washington.edu/research/hsd/docs/1253. Before answering the questions below, be sure that you are familiar with the definition.

1. **Subject groups/categories and numbers.** Complete this table by listing:

- Your groups or categories of subjects. "Group" should be defined as appropriate for your research.
  - "Units" within a group. For most research, a group will consist of individuals, such as children aged 8-12, or individuals with high blood pressure. However, this will not be true for all research. Examples of groups with "units" that are not individuals:

- Dyads such as Alzheimer's-patient-and-caregiver, with one group of the dyads assigned to one intervention (e.g., behavioral modification) and another group of the dyads assigned to a comparison intervention(e.g., drug treatment).
- Families. For example, a study of mental health interventions for homeless families might have one group of 30 families assigned to one intervention and another group of 30 families assigned to a different intervention.
- Other. For example, the "units" in autism research might be an autistic individual and all his/her living blood relatives. The units in an academic excellence study might be a student-parents-teacher unit.

o Types of groups. There are many ways in which subjects might be grouped. Examples:
- By intervention. Example: research comparing two different drugs for high blood pressure.
- By subject population. Example: research comparing the incidence of domestic violence in families living in urban settings versus families living in rural settings.
- If you have only one group, fill in only one line in the table. Add more lines if needed.

• The age range of each group.

• The upper limit/number of **completed** subjects you need for each group. ***Completed*** *means that all research procedures involving the subjects or the obtaining of specimens/records/data have been completed as far as is possible for each subject, including any follow-up (such as follow-up access to medical records.) In some cases, such as an online survey, it is not possible to predict the number of subjects who will complete the research. If you cannot predict or describe the maximum number of subjects you need in each group, check the appropriate box and provide your rationale in the space provided below the table.*

**Group name/description**	**Age range of subjects**	**Maximum desired number of individuals (or other group unit, such as families) who will complete the research.***	**Cannot provide a number.****
HIV-TB co-infected adults	≥ 18 years of age	36	☐**
			☐**
			☐**
			☐**

***This is the number of subjects (individuals, dyads, families, etc., as appropriate) in each group that will be considered for approval by the IRB.**

****If you cannot predict or describe the maximum number of subjects you need in each group:**

Provide your rationale and description of research scope here. *Include any information or estimates you might have about the number of subjects, so that the IRB has a sense of the scope of your research. For example, your research might be a small pilot study of all patients presenting with a rare disease at UW Medicine in the next year. Or, it might involve a survey posted on Craig's List for two weeks that could result in thousands of responses.*

NOTE: In your periodic Status Report, you will be asked to complete the table below with your subject numbers. While developing your research protocol, please plan ahead so that you will have an accurate record of the subject numbers above.

This is for illustration only. Do not complete this table.

Group Name / Description	# Completions				# Ongoing (subjects still involved)	# Withdrawals, drops, lost		
	Total approved by IRB	A At time of last Status Report	B Since last Status Report	A + B Total to date		C At time of last Status Report	D Since last Status Report	C + D Total to date

DO NOT COMPLETE

2. Explain how you will achieve equitable subject representation in the following categories. If not applicable, justify exclusions.

Patients ≥ 18 years of age will be enrolled. Methods for assessing nutritional status, food insecurity, and dietary diversity are different in minors compared to adults and appropriate nutritional interventions for children versus adults may be different. Therefore, minors (individuals < 18 years of age) will be excluded from this study. Males and females will be enrolled in an expected proportion of 1:2 based on the demographics of HIV in Senegal. These studies will be conducted in Senegal, therefore subjects will be Africans belonging to the ethnic/tribal groups found in Senegal.

3. What characteristics (inclusion criteria) must subjects have to be in this study? (Answer for each subject group, if different.)

   Individuals will be eligible for inclusion if they are 18 years of age or older, HIV positive, and initiating treatment for tuberculosis.

4. What characteristics (exclusion criteria) would exclude subjects who are otherwise eligible from this study? (Answer for each subject group, if different.)

   Individuals with a history of an allergy to any of the ingredients in the nutritional supplements will be excluded.

5. Describe the subject recruitment strategies you will use for each group of subjects. (When applicable, you should obtain letters of cooperation from agencies, institutions, or others involved in subject recruitment for your research records. Do not send these to HSD or the IRB.)

   Clinic charts will be reviewed (pre-screening) to identify potentially eligible subjects and clinic staff will be informed of study enrollment criteria. Potentially eligible subjects will be referred to the social worker.

6. Explain who will approach subjects to take part in the study and how this will be done to protect subjects' privacy.

   Potentially eligible subjects will be approached by the social worker in a private setting.

7. Explain what steps you will take during the recruitment process to minimize potential coercion or the appearance of coercion.

   Subjects will be informed that enrollment in the study is voluntary and that a decision not to enroll will be confidential and will not influence their clinical care.

8. Will you give subjects gifts, payments, services without charge, or extra course credit? ☐ No ☒ Yes If yes, explain:

Subjects will be reimbursed for the cost or transportation to study encounters (approximately 3 USD).

9. Will any of the subjects or their third-party payers be charged for any study procedures? ☒ No ☐ Yes If yes, explain:

10. **<u>UW Locations and research sites.</u>** Provide the following information in <u>list or table format</u> for all UW locations at which any research procedures will occur. Be sure to consider: screening, recruiting, consenting, observation, intervention, data collection, data analysis, specimen analysis, and location of any consultants and collaborators.

    - Geographical location and/or address
    - Name of organization, agency, group, site, institution
    - What procedures will occur at each location (how the location is involved in the research)
    - Whether subject contact or interaction will occur at each site
    - Whether consenting of subjects will occur at each site
    - Whether each site, or individuals at the site, will obtain, use, or have access to coded or individually identifiable private information about subjects for research purposes

Geographical location	Institution	Procedures	Subject contact on site	Consenting on site	Access to individually identifiable private information
Seattle, WA	University of Washington	Data entry and analysis. Manuscript preparation	No	No	Yes

E. RISKS AND BENEFITS

In order to approve the research the IRB must find that risks are reasonable in relation to anticipated benefits, if any, to subjects, and the importance of the knowledge that may reasonably be expected to result.

1. Describe nature and degree of risk of possible injury, stress, discomfort, invasion of privacy, and other side effects from all study procedures, drugs and devices (standard and experimental), interviews and questionnaires. Include psycho-social risks as well as physiological risks. Include risks of withholding standard care or procedures if this is the case. Do not reference the consent form.

   The primary risk of study participation is breach of confidentiality. There is a potential risk of causing emotional distress through discussion of sensitive topics. Approximately 30 ml of blood will be collected at the enrollment and 6 month follow-up visits. The risks associated with drawing blood include minor (if any) discomfort at the site of venipuncture, possibly a slight bruise or bleeding, and a very low risk of fainting and infection.

2. Explain what steps you will take to minimize risks of harm and to protect subjects' rights and welfare. (If you will include protected groups of subjects (minors; fetuses in utero; prisoners; pregnant women; unviable neonates; neonates of uncertain viability; decisionally impaired or economically or educationally disadvantaged subjects) please identify the group(s) and answer this question for each group. Please also complete the SUPPLEMENT: Protected and/or Vulnerable Populations.)

Risk of breech of confidentiality: PHI will be stored in secured offices and on password protected encrypted computers. Only study personnel will have access to PHI as needed to perform the study.

Risk of patient distress: Questions that may be interpreted as offensive or embarrassing will be avoided. Patients will be reminded that they can refuse to respond to any question or withdraw from the study at any time without consequence.

Risk of venipuncture**:** Sterile, single use blood draw equipment using aseptic technique and universal precautions by trained personnel will be performed.

3. Is it possible that you will discover a subject's previously unknown condition (disease, suicidal intentions, genetic predisposition, etc.) as a result of study procedures? ☐ No ☒Yes If yes, explain how you will handle this situation.

   If a previously unknown condition is identified, the subject will be referred to their clinician.

4. Describe the anticipated benefits of this research for individual subjects in each subject group. If none, state "None."

   The potential benefits to subjects include improvements in nutritional and food security status, increased adherence to tuberculosis treatment and ART, improved clinical outcomes through the indirect effects of being enrolled in a research study, and increased emotional/social support through monthly study encounters. It is also possible that this study will have no direct benefits for individual subjects enrolled in the study.

5. Describe the anticipated benefits of this research for society.

   This implementation study will provide practical information about how to optimally integrate nutritional supplementation into HIV and TB programs in Senegal. This has the potential to improve HIV and TB outcomes, decrease mortality, and improve quality of life.

F. ADVERSE EVENTS OR EFFECTS

1. Who will handle adverse events? ☐ Investigator ☒Referral ☐ Other, explain:
2. Are your facilities and equipment adequate to handle possible adverse events? ☒Yes ☐ No, explain:

G. CONFIDENTIALITY OF RESEARCH DATA

1. Will you record any direct subject identifiers (names, Social Security numbers, patient, hospital, laboratory or claim numbers, addresses, telephone numbers, locator information, etc.) ☐ No ☒ Yes If yes, explain why this is necessary and describe the coding system you will use to protect against disclosure.

   Patient names will appear on the consent forms. Names, birthdates, phone numbers, and addresses will be recorded in order to remind subjects of follow-up appointments and to contact those who are lost to follow-up. Clinic identification numbers will be recorded in order to perform chart review. Direct subject identifiers will be linked to study code numbers and stored in secured offices.

2. Will you retain a link between study code numbers and direct identifiers after the data collection is complete? ☐ No ☒ Yes If yes, explain why this is necessary and for how long you will keep this link.

   For subjects who have agreed to be contacted for future studies, data will be retained in linked form for 20 years. For subjects who do not agree to be contacted for future studies, data will be retained in linked form for 10 years.

3. Describe how you will protect data against disclosure to the public or to other researchers or non-researchers. Explain who (other than members of the research team) will have access to data (e.g., sponsors, advisers, government agencies, etc.).

   All PHI data will be stored in secured offices and on password protected encrypted computers. Only study personnel will have access to PHI as needed to perform the study.

4. Will you place a copy of the consent form or other study information in the subject's medical or other personal record? ☒No ☐ Yes. If yes, explain why this is necessary.

5. Do you anticipate using any data (information, specimens, etc.) from this study for other studies in the future? ☐ No ☒Yes If "Yes," explain and include this information in the consent form.

   Data from this study will be used to design a larger clinical trial of nutritional interventions among HIV positive individuals in Senegal. Specimens may be used for future biomedical research, including studies of HIV, TB, and other infectious diseases in Senegal.

H. ADDITIONAL INFORMATION

1. If the study will involve radiation exposure to subjects, e.g., X-rays, radioisotopes, what is status of review by the UW Radiation Safety Committee (RSC): ☐ Pending ☐ Approved (Attach one copy of approval.) ☒NA

2. Does this research require approval from the UW Institutional Biosafety Committee (IBC) for recombinant/synthetic DNA human Gene transfer or vaccines?

   ☒ No ☐ Yes. If yes, what is the status of review by IBC? ☐ Pending ☐ Approved (Attach one copy of approval.) ☐ NA

3. Protected Health Information (PHI). Will you or any member of your research team obtain, access, or use a subject's protected health information by any method, and for any purpose including "pre-screening"?

   "Methods" may include but are not limited to: directly looking at a medical record (electronic or paper), requesting medical record information from a service such as the UW Center for Health Excellence, or viewing surgery schedules, clinic records, appointment books, etc.

   Examples of where PHI may be located include: medical records, dental records, clinical lab tests that you will have performed on subject samples, pharmacy records, medical billing records, clinical databases, etc.

   ☐ No ☒Yes. If "yes":

   a. Describe the type of records/data, location and how you will obtain the information:

      Clinic medical records will be reviewed to identify potentially eligible subjects (pre-screening). Those who are enrolled in the study will undergo further review of their medical records in order to record details of their medical history.

   b. Will you obtain any of the information without HIPAA authorization from each subject?

   Not applicable. This study will take place in Senegal.

      ☐ No ☐ Yes. If "yes": Complete and attach the SUPPLEMENT: Waiver Request, HIPAA Authorization, and the SUPPLEMENT: Waiver Request, Consent Requirements. If the records are owned by the University of Washington or a state agency, complete and attach a UW Confidentiality Agreement.

   c. Will you obtain HIPAA authorization from subjects for any of the information?

      ☐ No ☐ Yes.

   d. Will you be obtaining any of the data as a Limited Data Set?

      ☒ No ☐ Yes.

4. Other Records. Will you or any member of your research team obtain, access, or use academic, employment, or any other type of records about subjects, by any method, and for any purpose including "pre-screening"?

   "Methods" may include but are not limited to: directly looking at a record (electronic or paper), requesting records from offices such as Payroll or the UW Registrar's Office, obtaining records from the state Department of Health, etc.

☒No ☐ Yes. If "yes":

a. Describe the type of records/data, location, and how you will obtain the information.

b. Will you obtain any of the information without the subject's consent?

☐ No ☐ Yes.

If the records are owned by the University of Washington, complete and attach a UW Confidentiality Agreement.

5. Will you use the Clinical Research Center (CRC) at the UW or Seattle Children's for any of your research activities?

☒ No ☐ Yes.

If you answered "yes":

A medical record will be created for your subjects at UW Medicine and CRC staff may need to access those medical records for you. This may be because they are performing procedures or collecting data for you. It may also be required if an event happens on the CRC that requires treatment (such as fainting during a blood draw). This means that you must obtain a signed HIPAA Authorization form from each subject and give a copy of it to the CRC.

6. Does your research involve any of the following:

- Students age 21 or younger who may be participants in your research?
- Access to, or use of, personally identifiable information from student (current or past) education records from any institution or agency of education (including, but not limited to, pre-elementary, secondary, post-secondary, job training, adult education, career and technical education, special education)?
- Conducting any research procedures in an educational setting?

☒ No ☐ Yes.

If you answered "yes":

Your research may be subject to the requirements of the **Protection of Pupil Rights Amendment (PPRA)** and/or the **Family Education Rights and Privacy Act (FERPA).**

Consult with the SOP Research Involving Students to determine whether PPRA or FERPA regulations apply to your research.

Check the appropriate box.

☐ PPRA regulations apply to my research

☐ FERPA regulations apply to my research

☐ Both PPRA and FERPA regulations apply to my research

☐ Neither set of regulations apply to my research

7. Will you make audio-visual or tape recordings or photographs of subjects? ☒No ☐ Yes. If yes, explain what type of recordings you will make, how long you will keep them, and if anyone other than the members of the research team will be able to see them.

8. Will your study involve use of equipment involving energy input to the subjects (EMG, EKG, MRI, ultrasound, etc.)? ☒No ☐ Yes. If yes, attach documentation that all equipment will be tested regularly by the Scientific Instrument Division (call (206) 543-5580 for information) or describe safety testing procedures you will use.

9. Confirm by checking the box that the principal investigator on this IRB application has ensured that all investigators (as defined by UW policy GIM 10) are aware of policy GIM 10 and their responsibility for complying with its relevant requirements.

☒ Confirmed

10. Does the individual who is the principal investigator on (1) this IRB application or (2) any grants or contracts supporting this research have a financial conflict of interest with respect to this research? ☒ No ☐ Yes.

If yes, has it been disclosed to the University? (Since August 24, 2012, all disclosures are made through the University's online Financial Interest Disclosure System.) Final review of this application cannot occur until the disclosure has been made and reviewed by the University, and the outcome has been incorporated into the IRB's review. ☐ No ☐ Yes ☐ Not applicable, because there is no financial conflict of interest.

11. Is your research:

- Clinical research that will bill subjects or their health insurance for UW Medicine professional or facility services, items, or tests*, **AND/OR**

- An "applicable clinical trial" as defined below" **

  ☒ No ☐ Yes

  If you selected "yes", you must register your research at the federal site ClinicalTrials.gov

  See the HSD document titled: ClinicalTrials.gov – Instructions for Registering Your Trials for step-by-step instructions about how to register your research.

  ***New Requirement**

  As of January 1, 2014, a new federal requirement will require you to provide the clinical trials registration number assigned to your research in order to bill most UW medicine professional or facility services, items, or tests to research participants or their health insurance. This new billing requirement applies to some clinical research, such as Phase I studies, that don't meet the federal registration definition of "applicable clinical trials". *See also*: Clinical Research Budget & Billing Support (CRBB)

  ***Applicable clinical trial is defined as:*

  (1) a pediatric postmarket surveillance study required by the FDA ***OR***

  (2) an interventional study (with one or more arm) of an FDA-regulated drug, biological product, or device that involves health outcomes and meets one or more of the following conditions:

  - *The trial has at least one site in the United states; or*
  - *The trial is conducted under an FDA investigational new drug application or investigational device exemption; or*
  - *The trial involves a drug, biologic, or device that is manufactured in the United States or its territories and is exported for research*

  The source of funding (e.g., industry, federal, nonprofit) is irrelevant.

See this website for additional information: http://prsinfo.clinicaltrials.gov/ElaborationsOnDefinitions.pdf

I. CONSENT

Obtaining informed consent is a process that involves more than obtaining a signature on a form. It is a process of information exchange that may include subject recruitment materials, verbal instructions, question-and-answer sessions, and measures of participant understanding. Obtaining voluntary informed consent is one of the central protections required by all human subjects regulations and ethical principles. The key features of the consent process include:

- Disclosure of the information needed to make an informed decision about participation
- Facilitation of comprehension by the potential participant
- Promotion of the voluntariness of the potential participant's decision

Refer to the **SOP Consent** and **SOP Consent Documentation** for more information.

1. Description of consent process for adult subjects. How are you going to obtain informed consent from your adult subjects? Describe in detail your consent methods, process, and settings. Identify who will provide the information to subjects and who will interact with them during the consent process. If there is more than one consent process, describe each one separately. For subjects who do not speak English: Describe the process that will be used, and whether anyone on the research team will speak the subjects' language. **Complete this section if you will obtain consent from any subjects for any aspect of the research.**

   Informed consent will take place in a private setting and will involve the subject, the social worker, and when applicable, an impartial witness. The written consent form will be provided in French. For subjects who can read French, the consent form will be provided to them directly. For subjects who cannot read French but understand French, the document will be read to them aloud in French. For subjects who cannot understand French, the consent form will be read to them aloud in a tribal language of Senegal that they understand (non-written languages, mostly Wolof, but may include Peul, Jola, Mandinka, Serer, Creole, etc.). Subjects will be encouraged to ask questions about the study. Subjects will indicate consent by providing their signature or written mark on the written consent form.

2. Description of assent process for children subjects. Describe in detail how you will obtain assent from children subjects, following the instructions provided in the question above. Also, describe how these processes will differ based on age/cognitive ability. Finally, describe how you will determine whether a child is assenting or dissenting throughout the

research (if applicable). **Assent means a child's affirmative agreement to participate in the research. Mere failure to object should not be interpreted as assent.*

Not applicable.

3. Special issues or considerations. The standard concept of consent is based on the Western ethical tradition of individual autonomy and privacy. This may not apply well to your research. Your research may be subject to specific cultural or other contextual issues that affect the consent process. Describe any special issues and considerations about obtaining consent for your research. If none, state: "Not Applicable".

   Example issues:

   - Who is the appropriate person(s) for providing consent?
   - The desirability of a group consent process, or a surrogate consent process
   - Research that occurs in a setting with a blurred sense of what is public versus private
   - The cultural acceptability of the consent process (or documentation)
   - Cultures or groups in which it is considered impolite to refuse a request and/or in which people are fearful of refusing requests that they regard as coming from authorities

   Not applicable.

4. Undue influence. Describe how you will minimize any undue influence on your subjects' decision about participating in your research. If this is not an issue for your research, describe why. *This is an important consideration when persons recruiting or consenting subjects are in a position of authority or influence – for example, the subject's teacher, doctor, or employer.*

It will be emphasized that participation in the study is entirely voluntary, a decision not to enroll will be kept confidential, a decision not to enroll will not influence clinical care, and that participants can decline further participation at any time.

5. Subject comprehension. Describe anything that you will do to facilitate or verify your subjects' comprehension of the information you provide them during the consent process.

   Subjects will be encouraged to ask questions or raise any concerns they have regarding study participation. They will be asked to reiterate their understanding of the study objectives and the potential risks involved.

6. Do you expect that all of your participants will be **fluent** in spoken and/or written English? ☒ No ☐ Yes.

   If "No", please answer the following questions.

   6.1. In what language(s) will they be fluent?

   Subjects will be fluent in French and/or a tribal language of Senegal (Wolof, Peul, Jola, Mandinka, Serer, Creole, etc.)

   6.2. Translation of documents into another language. Federal regulations require that consent, assent, and authorization documents must be presented to participants in a language that is understandable to them. The UW IRB expects that translated documents will be:

   - Linguistically accurate;
   - At an appropriate reading level for the subject population; and
   - Culturally sensitive for the locale in which they will be used.

   Describe how you will obtain translations of relevant documents, and how you will ensure that the translations meet these requirements.

   Consent forms will be translated by the PI and reviewed by the study coordinator who is fluent in French and English, and has experience translating consent forms for previous UW studies conducted in Senegal.

   6.3. Interpretation. Describe how you will provide interpretation, and when. Specifically:

   a. For what situations will you provide interpretation? (At a minimum, an interpreter should be available for the consent process, unless the IRB has waived consent.)

      Interpretation will be available during the consent process, enrollment, and all subsequent study encounters.

   b. Who will be the interpreter?

      The Centre de Santé de Ziguinchor has interpreters available on site.

c. Describe the qualifications of the interpreter – for example, background, experience, language proficiency in English and in the other language, native language fluency, certification, other credentials, familiarity with the research-related vocabulary in English and the target language.

Interpreter qualifications will include a basic understanding of English, fluency in French, and fluency in the local languages of the region (Wolof, Peul, Jola, Mandinka, Serer, Creole, etc.).

d. How will you ensure that the subjects will understand ongoing study-related communication? If the subject has questions, complaints, or adverse events, how will that be communicated to the researchers?

Subjects will be instructed to report any concerns to the study coordinator.

7. Check all that apply:

☒ **Written** *Attach copies of all consent forms for each subject group. Include a footer identifying the version date of each form and a header or title that identifies each different form. If you propose to delete one or more of the required elements of consent from a consent form, attach and complete the form called SUPPLEMENT: Waiver Request, Consent Requirements.*

☐ **Waiver of written documentation of consent** *This means that you are requesting a waiver of the requirement to obtain written documentation of consent. Complete and attach the form called SUPPLEMENT: Waiver Request, Consent Requirements. . Also, attach the Information Statement, oral consent or assent protocol and script, or other materials you will use to communicate the necessary elements of consent to the subjects.*

☒ **Waiver of consent for the purpose of pre-screening only.** *This means that you are requesting a waiver of the requirement to obtain consent. Complete and attach the form called SUPPLEMENT: Waiver Request, Consent Requirements. .*

☐ **Assent** *Attach copies of any written materials or scripts you will use with minor subjects (individuals under the age of 18) to obtain their assent to being in your research.*

☐ **Parental permission** *Attach copies of any written materials or scripts you will use with parents, to obtain their permission to enroll their minor children in your research. See also SUPPLEMENT: Protected and/or Vulnerable Populations for waivers or alterations of consent requirements.*
